# Laser fistula treatment: beyond the controversial aspects: best clinical practice recommendations from an international group of surgeons with extensive experience in the procedure−the FiLaC recommendations

**DOI:** 10.1007/s10151-025-03164-w

**Published:** 2025-06-09

**Authors:** P. C. Ambe, G. P. Martin-Martin, A. A. Alam, S. Chaudhri, B. Bogdanic, H. Ma, B. Bolik, I. H. Roman, J. Wu, J. D. P. Hernandez, N. Vasas, Q. Dong, P. Istok, R. Schouten, S. Kalaskar, Y. Yao, T. Bruketa, E. Koulouteri, V. Dobricani, C. Zhe, P. Giamundo

**Affiliations:** 1 Faculty of Medicine, Witten/Herdecke University, Witten, Germany; 2 Department of Surgery, Klinik Oberwart, Oberwart, Austria; 3 Hospital Doctor López-Cano, Cádiz, Spain; 4 Department of Coloproctology of Saint Joseph Hospital of Paris, Institute of Leopold Bellan, Paris, France; 5 Consultant Colorectal Surgeon, University Hospitals of Leicester, Leicester, UK; 6 Department of Surgery, University Hospital Centre Zagreb, School of Medicine, University of Zagreb, Zagreb, Croatia; 7 Editorial Department of Journal of Colorectal and Anal Surgery, The First Affiliated Hospital of Guangxi Medical University, GuangXi, China; 8 St. Elisabeth-Hospital Bochum, Bochum, Germany; 9 Department of General, Visceral Surgery and Coloproctology, Clinique CIC Suisse-Vivalto Santé, Montreux-Clarens, Switzerland; 10 Department of Coloproctology, Yueyang Hospital of Integrated Traditional Chinese and Western Medicine, Shanghai University of TCM, ShangHai, China; 11 Cirugía General y Digestive, HU Ramon y Cajal, Madrid, Universidad Alcalá de Henares Clínica Grupo Pedro Jaén, Madrid, Spain; 12 Kardirex Medical Center, Gyor, Hungary; 13 Longhua Hospital Shanghai University of Traditional Chinese Medicine, Shanghai, China; 14 Proktovena, Brastislava, Slovakia; 15 Department of Surgery, Flevoziekenhuis, Almere, The Netherlands; 16 Department of General Surgery, Dartford & Gravesham NHS Trust, Dartford & Gravesham, UK; 17 Department of Coloproctology, LongHua Hospital Shanghai University of TCM, ShangHai, China; 18 General Surgeon Limassol, Limassol, Cyprus; 19 Department of Surgery, Clinical Center of Montenegro, Podgorica, Montenegro; 20 Renji Hospital, Shanghai Jiao Tong University School of Medicine, Shanghai, China; 21 Department of Surgery, Città di Bra Clinic, Bra, Italy

**Keywords:** Fistula tract laser closure, FiLaC, Fistula in ano, Anal fistula, Sphincter preserving fistula surgery, Laser Proctology

## Abstract

**Background:**

Fistula tract laser closure (FiLaC) represents a minimally invasive, sphincter-sparing technique for managing fistula in ano with increasing popularity among proctologists. Despite its increasing adoption, significant variations exist in the application of FiLaC in daily practice.

**Purpose:**

The aim of these recommendations was to define some basic principles and recommendations for performing a standard FiLaC procedure.

**Methods:**

The recommendation development group (RDG) consisting of surgeons with experience in the FiLaC were invited to formulate recommendations for the procedure. The recommendations were generated following systematic literature research and discussion amongst experts (expert opinion) where no substantial literature was available. The developed recommendations were voted upon by a panelist via the Delphi process. Consensus was a priori defined as agreement of 75% and above.

**Results:**

The RDG developed 25 recommendations that were voted upon by 21 panelists from 13 nations. Consensus was reached for all 25 recommendations after the first Delphi round.

**Conclusion:**

The FiLaC RDG offers a comprehensive suite of recommendations to enhance the safety and efficacy of standard FiLaC procedures. These 25 detailed recommendations collectively address the full spectrum of FiLaC procedures—from laser settings, preoperative preparations, and perioperative strategies to postoperative care. This coherent framework is anticipated not only to standardize but also to refine the FiLaC technique to ensure best possible surgical outcomes while preserving patient safety.

## Introduction

Anal fistula represents a significant surgical challenge, with an estimated annual incidence of 1.2–2.8 cases per 10,000 individuals in Europe, predominantly affecting men aged 30–50 years [1, 2]. Classic treatment techniques include fistulotomy, advancement flaps, the LIFT procedure (ligation of the intersphincteric fistula tract), and fistulotomy with immediate sphincter reconstruction [3–5]. Fistulotomy is widely used and offers high healing rates for simple fistulas, although its use in complex fistulas is associated with a higher risk of incontinence. Advancement flaps, often indicated for high or complex fistulas, show success rates ranging from 60% to 80%, depending on factors such as smoking and fistula tract anatomy. The LIFT procedure has proven effective in selected cases, with healing rates above 70%, but with limitations in general applicability [6]. Laser ablation of anal fistulas was first described by Arne Wilhelm in 2011 as an innovative technique in the minimally invasive management of this condition [7]. Using radially emitting laser probes, such as the FiLaC^®^ (Fistula-tract Laser Closure) system, this technique enables controlled destruction of the fistula tract epithelium, leading to obliteration and closure of the fistula tract. The 1470 nm wavelength used in this system ensures limited penetration into the tissue, minimizing the risk of sphincter damage and preserving anal function [7]. According to a recent meta-analysis, the weighted mean primary healing rate with FiLaC was 67.3%, with an overall success rate of 69.7% after repeating the procedure following initial failure. Moreover, minimal complication rates of about 4% and incontinence affecting only ca. 1% of cases have been reported in recent literature [8]. Long-term results show that at a median follow-up of 60 months, the primary success rate was about 66.8%, while a secondary success rate of up to 73.7% was achieved with repeat procedures [9]. The FiLaC method presents itself as a promising and safe alternative for the treatment of anal fistulas. Its minimally invasive nature aims to preserve continence and reduce the surgical trauma associated with more conventional techniques. Furthermore, the homogeneity of the energy emitted by the laser facilitates a controlled and reproducible application, offering potential for standardizing its use in the management of this pathology. This work aims to establish recommendations based on expert opinions through a Delphi process, laying the groundwork for its appropriate clinical implementation. 

## Methods

The need for a standardized protocol for laser procedures was initiated during the PROCTOCOM Expert meeting in Malaga/Spain in June 2023 as reported previously [10]. This meeting was organized by Biolitec Biomedical technology GmbH, Jena, Germany, a producer of laser solutions, as part of a continuous quality improvement, training and communication amongst surgeons offering laser interventions in coloproctology. Large heterogeneity amongst surgeons performing FiLaC was identified, and the need to standardize FiLaC was deemed necessary [11]. In the next step, experts in the FiLaC procedure were invited to an introductory video meeting to discuss the need for treatment recommendations for FiLaC. A face-to-face meeting took place during the 2024 ESCP annual meeting in Thessaloniki.

As a result of a lack of high-quality publications, the Delphi method was deemed most effective, especially to counteract the effect of expert opinion while formulating recommendations [12, 13]. It was agreed upon a priori that at least 75% agreement is necessary for consensus [14, 15]. Also, a maximum of three voting rounds was defined a priori [16, 17], each round lasting 14 days. Statements with at least 85% agreement would be declared “strong consensus” [18, 19].

Systematic literature research for available publications related to FiLaC was performed using a search strategy comprising combinations of the following terms: FiLaC, fistula laser tract closure, laser fistula closure, LAFT, laser assisted fistula closure, laser assisted fistula tract ablation, laser ablation of anal fistula. The search was limited to articles published in English language up to July 2024. Case reports, experimental studies, technical papers, conference papers, and narrative review articles were excluded. The references of included articles were consulted for possible additional publications. The articles generated by the search strategy were included in a “FiLaC library” which was made available to the panelists. All participants were encouraged to send in any publications that were not part of the FiLaC library.

In the next step the panelists were asked to send in questions and comments on all aspects of FiLaC after studying the FiLaC library and based on their clinical experience with FiLaC. Equally, members of the steering committee (academic surgeons with extensive experience in FiLaC) were invited to identify and submit questions and controversial issues regarding the FiLaC procedure based on their profound clinical experience and on the currently available literature. All submitted questions and comments were scanned by members of the steering committee and used to formulate the Delphi questions (DQ).

Answers to the DQ were suggested and discussed by members of the steering committee after appraising the available literature regarding the level of evidence [20, 21]. Each DQ was commented upon using available publications. Discordances amongst experts were resolved by open discussions moderated by the project leader PCA. Finally, a clear response to each DQ was formulated. The completed DQs with corresponding responses (Rs) and the accompanying commentaries were discussed once more in the steering committee with minor corrections and/or rephrasing before being cleared for the Delphi rounds.

The online tool Zoho CRM Plus Survey was used for the Delphi process [22]. The DQs and their corresponding responses, commentaries, and references were uploaded on the server and a link to the survey was generated, which was mailed to all panelists for voting. Voting stopped 14 days after initiation and the link was deactivated.

## Results

The project was initiated in September 2023 and the first introductory video meeting was held on September 14, 2023. At the beginning, 90 identified experts in laser proctology were invited to join the project. Following the second and third invitations, 50 international experts responded, signaling interest to participate, including 21 that were involved in the project until its completion. These 21 panelists from 13 nations constituted the recommendation development group (RDG); five from China, two from Germany, Spain, UK, and Croatia, as well as one participant from Italy, the Netherlands, France, Cypress, Hungary, Slovakia, Montenegro, and Switzerland.

**Fig. 1:**
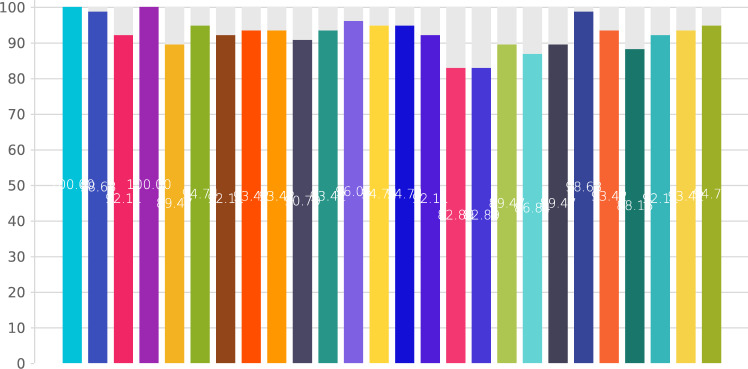
Results of the first Delphi round

The RDG defined 25 DQs (Q1–Q25) concerning FiLaC which were discussed and commented upon based on the current literature and expert opinion, resulting to the generation of 25 responses (R) or Delphi statements. The 25 DQs and commented statements were voted upon by 21 panelists. All 25 items scored above 75% in the first Delphi round; thus, voting was terminated (Fig. 1).

### Q1: What are the minimum requirements in laser surgery and is a workshop attendance mandatory?

Laser surgery in proctology has rapidly evolved and many surgeons across Europa and Asia are adopting this new technique [23]. The need for a formal training in laser surgery has so far not been addressed. It is common sense that the outcome of any surgical procedure largely depends on the expertise of the operating surgeon. This simple rule holds for laser surgery. Although no data has been published on this aspect of FiLaC, the RDG uniformly agrees on the need for a well-defined training program for laser-assisted proctological procedures, especially FiLaC. The RDG is very clear on the fact that an observation alone is not enough and that at least one workshop with optimally hands-on training represents a minimum requirement for surgeons prior to performing FiLaC. Therefore, besides being trained in the management of coloproctological pathologies and having experience in the management of fistula in ano, the completion of at least one FiLaC workshop represents the minimum requirements for performing FiLaC. The RDG encourages mentoring of surgeons during their first cases of FiLaC or direct training with a mentor.

R1: *Besides being a trained proctologist, completion of at least one workshop, optimally with hands-on training, represents the minimum requirements prior to performing FiLaC. Active mentoring of the first FiLaC cases is very much encouraged.*

### Q2: Which laser wavelength should be used for FiLaC?

The current literature is inconsistent about the laser settings for FiLaC. The most used wavelengths currently in laser proctology are 980 nm and 1470 nm. The 980 nm wavelength has been reported in only a few series for FiLaC. Giamundo et al. used the 980 nm wavelength to manage the first eight FiLaC cases in their primary publication on this technique in 2014 and subsequently switched to the 1470 nm wavelength for the same indication [9, 24, 25]. The current literature is vastly homogenous on the use of 1470 nm [9, 25–30]. Although these are all retrospective studies, they are all based on the 1470 nm wavelength. Thus, the RDG recommends that FiLaC should be performed with 1470 nm wavelength.

R2: *The 1470 nm wavelength is recommended for FiLaC.*

### Q3: How much power (watts) is appropriate for FiLaC?

The current literature is rather heterogenous with respect to the amount of laser energy used during FiLaC. In the first publication on FiLaC by A. Wilhelm, the power of the 1470 nm emitting laser was set at 13 W [7]. Since then, a variety of power settings have been reported in the literature. Low power of 10 W was used by some authors [31, 32], while other groups reported using 12 W [30, 33–35] and 13 W to perform FiLaC [28, 36, 37]. The outcome of FiLaC depends, at least to some degree, on the amount of laser energy used [38]. Low energy on one hand may be insufficient to successfully close the tract. Two studies using 10 W by Stijns et al. and Bhushan and Joshi reported very low healing rates of 20% and 40%, respectively. Very high energy on the other hand leads to carbonization of the epithelium with increased risk of treatment failure. On the basis of the available literature, the RDG unanimously recommends 12 W as the standard power for FiLaC. Alternative power settings may be chosen as needed on the basis of the surgeon’s expertise.

R3: *12 W is recommended for standard FiLaC.*

### Q4: What kind of fiber is optimal for/in performing FiLaC?

The literature is very homogenous in this regard. All publications on FiLaC reported the use of a 360° emitting fiber. Therefore, the fiber for FiLaC must be a 360° emitting ring-like fiber with light guidance. Various names have been used in the literature to describe this fiber, e.g., radial fiber [39, 40] or bare fiber [41]. FiLaC should be performed under visual guidance via the indicator light of the fiber, irrespective of the method of fiber introduction, i.e., with or without the aid of a seton.

R4: *A 360° emitting ring fiber should be used in performing FiLaC.*

### Q5: What is the role of tactile feedback or ultrasound/MRI images in setting the energy for FiLaC?

There is hardly any data looking at the relationship between findings from preoperative imaging, either by MRI or via endoanal ultrasound (EAU) and the amount of energy used during FiLaC in the current literature. Preoperative imaging may help make the decision on the timing of FiLaC regarding the size of the fistula tract, the presence or absence of accessory tracts and inflammatory collections, supported by clinical judgement [42]. See comment on seton (changing the caliber of the fistula tract into a FiLaC-friendly tract, Q9 and Q10).

Tactile feedback, especially regarding the fiber adhering inside the tract, should not be very relevant in FiLaC. According to expert opinion, the fiber may not always adhere to the wall of the tract. If, however, the fiber gets stuck, withdrawal must be done in a very gentle and careful manner, to prevent leaving back islands of untreated tract secondary to a forceful fiber withdrawal. The same caution should be exercised about re-intubation of the fistula tract. Equally, there is no data on this aspect of FiLaC. Proponents of this maneuver suggest a gentle teasing rather than a forceful re-intubation trial, because of the risk of reopening the sealed tract. The expert opinion is that the energy used in FiLaC should be independent of findings from preoperative imaging and tactile feedback.

R5: *The energy used in FiLaC should be independent of findings from preoperative imaging and/or tactile feedback.*

### Q6: Which type(s) of fistula according to Park’s classification can be treated with FiLaC?

FiLaC belongs to the spectrum of sphincter-preserving techniques for the management of fistula in ano [43–45]. Thus, all fistula types that may compromise sphincter function may be managed with FiLaC. Park’s classification may not be an appropriate tool on which to base the indication for FiLaC. From a clinical standpoint, the etiology of the fistula may become a relevant aspect to consider. Thus, the RDG looked at cryptoglandular fistulae, Crohn’s fistula, and rectovaginal fistula. On the basis of this, indications for FiLaC for cryptoglandular fistula may include high fistulae with either a transsphincteric or suprasphincteric course [46]. Besides, cryptoglandular fistulae in female patients independent of fistula type based on Park’s classification, as well as fistulae in patients with prior compromised sphincter function, represent good indications for FiLaC. A huge advantage for FiLaC compared to other sphincter-sparing procedures is that there is hardly any damage to the sphincter following FiLaC, even if the procedure fails. Also, the RDG strongly recommends FiLaC for all forms of perianal Crohn’s fistula [26, 47, 48]. However, in this group, FiLaC should not be performed in patients with active intestinal disease and proctitis.

A good argument for the use of FiLaC in high fistulae, e.g., high transsphincteric (Park’s type 3) and suprasphincteric (Park’s type 4) fistulae, is the possibility of downgrading the fistula tract to a less complex fistula, which can then be easily managed. In the paper published by Wilhelm et al., distalization (downgrading) of the fistula was seen in 50% of cases with treatment failure following FiLaC for high fistulae [29]. This interesting finding was also reported by Giamundo et al. [9, 24]. Irrespective of the type of fistula, the RDG highly recommends ruling out a diverticulum at the apex of the fistula tract in patients with supralevator and suprasphincteric fistulae, which may largely be responsible for treatment failure in such cases.

R6:* Cryptoglandular fistulae with a risk of postoperative compromise of the continence, Crohn’s perianal fistulae, and fistulae in female patients represent possible indications for FiLaC.*

### Q7: What are the contraindications for FiLaC?

Acute perianal inflammation with collections represents an absolute contraindication for FiLaC (see commentary on seton, Q9 and Q10) [49]. Also, simple fistulae, although not formally regarded as a contraindication for FiLaC, have higher healing rates with low risk of sphincter injury following fistulectomy. FiLaC may not work well in cases with active Crohn’s disease, both intestinal inflammation and proctitis. Although there are no publications on the use of FiLaC in rectovaginal fistula, the RDG sees rectovaginal fistulae as a contraindication for FiLaC. The expert opinion is that the probability for success is lower due to the relatively short fistula tract and possibly low tissue density over the anterior aspect of the tract.

R7: *Acute cellulitis and inflammatory collections or abscess formation, active Crohn’s, and rectovaginal fistula represent contraindications for FiLaC.*

### Q8: Could FiLaC be a first-line treatment?

The healing rate and the risk of continence disturbance represent two relevant outcome measures following fistula closure [50]. This is especially true regarding the management of complex or high fistula with involvement of a relevant portion of the sphincter muscle. The current literature suggests that healing rates of FiLaC (ca. 63–75%) are comparable with those of the currently used sphincter-sparing techniques for fistula closure. Moreover, FiLaC shows better results with respect to postoperative complications, especially continence disturbance [51]. Therefore, FiLaC has a place as a first-line option in fistula surgery. This is in line with the Danish publication by Nordholm-Carstensen et al. that reported FiLaC as a first-choice treatment [28].

For simple fistula, where there is hardly any risk of continence disturbance following conventional surgical techniques, lying the fistula open is associated with high healing rates [52, 53]. This outcome is better than the data for FiLaC [8]. Thus, FiLaC may not be a first-line option in the management of such cases. Moreover, factors to be considered in the decision-making, e.g., sex, fistula location (anterior vs. posterior), prior surgeries, and low resting tone, may tip the scale towards FiLaC in some cases with simple fistula. Also, other well-known advantages of FiLaC, e.g., less pain, smaller wound, early return to work, should be discussed with the patient.

R8:* FiLaC is one of the sphincter-sparing treatment options for patients with complex fistula and is therefore a first-line treatment option. FiLaC can be offered to selected patients with simple fistula in case of risk factors for postoperative continence disturbance.*

### Q9: Should a draining seton be used prior to FiLaC and for how long?

Drainage of any collection and management of cellulitis represent the initial treatment for patients presenting with anorectal abscess with or without fistula [54, 55]. The use of a draining seton has been shown to improve the healing rates following FiLaC [9]. A statistically significant higher healing rate was reported by Giamundo et al. in the group with draining seton vs. no draining seton (70.4% vs. 51.5%) [9]. As is the case with other fistula closure procedures, the use of a draining seton is strongly recommended [6]. The duration of the draining seton has not been well defined in the literature. For example, some leave the seton in place for 2 months [28]. The expert opinion is to keep the seton for at least 2 months. Thereafter, the timing of FiLaC could be determined based on both clinical judgement and findings from imaging (MRI and/or EAU).

R9:* A draining seton is recommended for at least 2 months. Thereafter, the timing of FiLaC can be decided upon based on clinical judgement and findings from imaging.*

### Q10: What to do if there is still purulent secretion despite seton placement?

Copious purulent secretion after a reasonable period (e.g., 4–6 weeks) following seton placement should prompt further investigation. Common reasons for this event could be an occlusion of the fistula tracts e.g., by the knot on the seton, if this is too large, or secondary to undrained accessory tracts. Large amounts of secretion in patients with Crohn’s fistula may be suggestive of proctitis [56, 57]. Such patients need an endoscopic evaluation and if needed medical optimization to achieve mucosal healing prior to FiLaC. Patient education and counseling about wound care including documentation (e.g., in a diary) of the amount of secretion from the fistula are recommended. Definitive surgery (FiLaC) should be postponed and the need to perform any kind of imaging should be made following clinical judgement.

R10:* FiLaC should be postponed in case of copious purulent secretion.*

### Q11: What is the role of imaging? Should MRI or EAU always be performed before FiLaC?

The timing of fistula closure has been shown to have a crucial effect on healing rates. This is especially the case regarding the cleanliness of the fistula tract. Traditionally, the absence of pus, associated with minimal secretion, may be indicative of a good time to perform fistula closure. While these clinical judgements remain unquestionable, the use of imaging has evolved as a meaningful adjunct to clinical judgement. Preoperative imaging with either MRI [58, 59] or EAU [60, 61] represents a standard aspect of preoperative workup prior to fistula closure. This is not different in the case of FiLaC (see comments to Q9).

R11:* Preoperative imaging either with MRI or EAU is strongly encouraged prior to FiLaC.*

### Q12: Is preoperative bowel preparation necessary? If so, what kind of preparation?

The need for bowel prepping and the kind of prepping to perform prior to FiLaC have not been systematically analyzed in the current literature. While some authors completely omit bowel prepping [30], using an enema to empty the rectum has been suggested by some surgeons [28, 62]. Opponents to enema see soiling secondary to enema as a problem. However, a clean rectum secondary to prepping is usually of advantage when there is a need to change the procedure, e.g., to advancement flap. According to the RDG, the decision to prep the bowel or not is at the discretion of the individual surgeon because this aspect of the procedure does not affect healing rate.

R12:* Bowel prepping prior to FiLaC can be omitted or performed based on surgeon’s discretion.*

### Q13: What is the role of pudendal block during FiLaC?

Postoperative pain following fistula surgery represents a relevant outcome measure and thus addressing this outcome is of interest. A major advantage of FiLaC in comparison with other surgical techniques is the reduced pain associated with the laser procedure [37]. Many studies in the current literature used either general or spinal anesthesia [27, 63]. In such cases, pudendal block in combination with general and regional anesthesia seems irrational. On the other hand, pudendal block could be a good add on to local anesthesia. The RDG voiced some reservation regarding the use of pudendal block in a potentially infectious condition (despite draining seton), especially looking at the expected low pain level after FiLaC. Therefore, the need for pudendal block should be well chosen and should consider the type of anesthesia for any individual patient.

R13: *Pudendal block may be omitted in patients undergoing FiLaC in general and regional anesthesia, and should be considered as an adjunct to local anesthesia or based on surgeon’s expectation of pain.*

### Q14: What to do when the fistula tract is too wide?

The principle of fistula treatment with FiLaC is based on the use of laser energy to denature the epithelized fistula tract, which then collapses and closes. Therefore, the results of FiLaC largely depend on the amount of energy that gets onto the wall of the fistula tract [24, 31, 38]. The probability that enough laser energy to achieve closure would reach the wall of a large tract is small. Therefore, the risk of failure in such cases may be high. Expert opinion is that the caliber of the fistula tract will usually adapt to that of the draining seton. However, this adaptation may take some time. A persistently large tract should warrant a clinical control and imaging in selected cases. Importantly, the patient should be counseled about the need for prolonged drainage in such cases. According to the RDG downsizing the seton may be a reasonable next step after waiting for about 3–6 months.

R14:* A persistently large fistula tract should prompt a downsizing of the draining seton.*

### Q15: What is the role of cochleation (coring out) and curettage during FiLaC?

Coring out a fistula has been shown to be a very good treatment strategy with high healing rates [64]. However, the involvement of the sphincter limits the extent of cochleation. Coring out the external fistula tract, however, represents an important step in FiLaC. This procedure basically widens the external opening to enhance drainage. Therefore, chochleating (coring out) the external opening is strongly recommended during FiLaC.

Performing a curettage of the fistula tract has been reported by some groups [26, 62]. However, this aspect of the FiLaC procedure is far from being standard. This is also true for fistula tract irrigation [63] as well as the combination of curettage and irrigation [32]. The current literature does not provide sufficient data to support either form of manipulation. The RDG, however, expressed concern about widening the fistula opening secondary to curettage. Furthermore, bleed/blood secondary to curettage may absorb part of the laser energy, thereby reducing the efficacy of the procedure. Should curettage be performed, achieving optimal hemostasis and flushing out clots is strongly recommended by the RDG. Therefore, while coring out the external opening is strongly recommended, irrigation and/or curettage can be performed at surgeon’s discretion.

R15:* Coring out (cochleating) the external opening during FiLaC is strongly recommended. Curettage and irrigation can be done at the surgeon’s discretion.*

### Q16: Can FiLaC be combined with other techniques?

While FiLaC has largely been used as a stand-alone procedure, many studies have reported a combination of FiLaC with other techniques. In the initial publication by Wilhelm et al., FiLaC was done in combination with advancement flap [29]. Since then, the use of some form of flap during FiLaC has also been reported by other authors [32, 63].

Recently, some studies on the combination of FiLaC with video-assisted anal fistula treatment (VAAFT) and ligation of the intersphincteric tract (LIFT) have been published [65, 66]. Some authors indicate the advantage of VAAFT in the visualization of the fistula tract with identification of accessory tracts, which obviously would predispose to treatment failure or recurrence. However, the role of cofounders should always be considered when many different techniques are used simultaneously to manage the same condition. Moreover, it remains questionable to what degree each procedure contributed to healing. Besides, one major advantage of FiLaC, low pain, may be scarified following the addition of other surgical techniques to FiLaC. According to the RDG, adding other procedures to FiLaC, besides closure of the internal opening (see Q17) and widening of the external opening should not be part of the standard FiLaC procedure.

R16:* The standard FiLaC is a stand-alone procedure.*

### Q17: Are the shape and diameter of the internal ostium relevant for closure?

The size and shape of the internal opening have not been widely investigated with regard to FiLaC. An analysis of 51 failed FiLaC cases by De Bonnechose et al. indicated a significantly higher failure rate of 84.6% in 16 cases with wide internal orifice compared to 50.6% in 41 cases with a narrow internal opening [67]. While this finding suggests that a wide internal opening may be a risk factor for failure, its clinical meaning is limited by some possible flaws in the surgical technique used by the authors. First, curettage of the fistula tract, which was performed in this study, may have widened the size of the internal opening. Second, the size of the internal opening was subjectively estimated by the operator, and finally the internal orifice was not closed. The expert opinion is that the shape and size of the internal ostium probably may not affect healing rate as this can be closed using a simple Z-stitch (figure of eight suture). In certain instances, the internal orifice is of such substantial size that achieving closure using a simple Z-stitch may present a significant challenge. This scenario is exemplified by highly inflammatory fistulas in Crohn’s disease with a very large internal orifice. An advancement flap can serve as a viable alternative to the simple Z-stitch, and when employed in conjunction with the FiLaC technique, it can yield optimal results.

R17:* The size and shape of the internal option should not influence FiLaC. However, in some cases, a very large internal orifice may require more than a simple stitch. *

### Q18: Should the internal opening be excised?

Excision of the internal opening during FiLaC has so far not been reported in the literature. According to the RDG excising the internal opening bears a risk of increasing the diameter of the fistula tract at this point as well as bleeding. These two complications may negatively affect the efficacy of FiLaC. Moreover, closure of a wide internal opening following excision may be more challenging.

R18:* Excision of the internal opening is not recommended.*

### Q19: What is the role of closing the internal opening and what is the recommended closure method/technique?

Whether to close the internal opening of the fistula track during FiLaC or not is a topic of discussion. While some authors did not close the internal opening [35], or did so only in individual cases [34], closing the internal opening has been used by many authors as part of the FiLaC procedure [8, 32, 63]. The technique employed in closing the internal orifice, however, has been very heterogenous. While some authors used a simple stitch (Z-stitch or figure of eight suture) [30, 36], flaps were constructed and used to close the internal opening by some others [29, 32].

In a study by Giamundo and De Angelis [9], the healing rate was higher following the closure of the internal opening (74% vs. 66%). Although this aspect of the surgical technique has not been systematically analyzed, closing the internal opening seems to be a relevant determinant for healing. While the RDG acknowledges this finding, closing the internal opening, especially using a flap, represents a definitive closure technique on its own. Therefore, it would be impossible to tell which technique (FiLaC or flap) contributed to what degree to the successful closure. Besides, extensive additive procedures like flaps may marginalize the low pain level following with FiLaC. Expert opinion of the RDG, therefore, is to close the internal opening with a simple stitch.

R19:* Closure of the internal opening with a simple stitch is recommended.*

### Q20: What is the optimal withdrawal speed for FiLaC?

The speed of withdrawal of the bare fiber is equally as crucial as the energy setting of the laser machine. A very high withdrawal speed may result in inlands of untreated tract epithelium while a very slow withdrawal speed may lead to carbonization of the tract. Both extremes may lead to treatment failure. Therefore, the speed of withdrawal is a relevant determinant for healing. Withdrawal rates of 1 mm/s, 1 cm/3 s, and 1 cm/6 s have been reported in the literature [25, 30, 33, 68–70]. A withdrawal rate of 1 mm/s has been the most reported rate and was adopted by the RDG as the most appropriate withdrawal rate.

R20:* A withdrawal rate of 1 mm/s is recommended for FiLaC.*

### Q21: What is the role of compression and packing of the external opening?

There is clear data from related subjects, especially following perianal abscess excision and drainage [71]. Compression and packing is associated with pain and discomfort, and has no significance in the healing process [72, 73]. These data can be easily extrapolated to FiLaC. If the need for compression and packing is to achieve hemostasis, then other methods to reach the same goal without packing/compression should be used.

R21:* Compression and packing are not recommended after FiLaC.*

### Q22: How should treatment success be evaluated?

Spelling out core outcome parameters to define healing after fistula surgery remains a topic of discussion [74]. The RDG suggests using clinical parameters, e.g., closure of internal and external opening and lack of discharge as well as patient-reported outcome measures like lack of symptoms of fistula (e.g., pain, swelling, soling, and discharge) to primarily define treatment success. These could be complemented by imaging, e.g., MRI in selected cases based on clinical judgement and the availability of resources.

R22:* Treatment success is primarily defined using clinical features and lack of symptoms, and imaging as needed.*

### Q23: What are the outcome criteria for FiLaC?

Accepted outcome criteria for FiLaC should not differ from well-established outcome criteria for other fistula repair procedures [74]. These parameters should include postoperative pain, healing rate, recurrence rate, rate of morbidity, especially incontinence rate, and quality of life amongst others. Healing and failure rates have been reported in almost all published manuscripts so far. However, wide heterogeneity exists amongst various publications reporting on healing rates. A systematic review of seven studies from European countries published between 2014 and 2018 including 454 patients reported a primary healing rate of 65.2% [8]. A more recent systematic review by Frountzas et al. included eight studies with 476 patients who underwent FiLaC, reported healing rates ranging from 40% to 89%. Interestingly, all kinds of fistula in ano including both simple and complex cryptoglandular fistulae as well as cases with Crohn’s fistulae were included in this systematic review [51]. A specific appraisal of the etiology of the fistula on the healing rate was highlighted by Cao et al. in 2022 after looking at the efficacy of FiLaC in patients with Crohn’s fistula. The authors performed a systematic review of six articles published between 2015 and 2021 in Europe including 50 patients with Crohn’s disease, showing a primary healing rate of 62% [47]. These results suggest that possible cofounders like etiology (Crohn’s vs. cryptoglandular), severity of disease (simple vs. complex fistula), preconditioning (draining seton), surgical technique (closure of internal opening, laser setting, and withdrawal rate), etc. should be considered when looking at the healing rates.

Postoperative pain represents a relevant outcome measure for patients undergoing interventions in proctology. The low pain level following FiLaC therefore represents a relevant postoperative outcome measure for patients. In a retrospective analysis by Marref et al. from the Groupe Hospitalier Paris Saint–Joseph, postoperative pain was reported using the visual analog score (VAS) as insignificant (VAS < 3) [37]. Interestingly, even in case of incomplete healing, some authors have reported a significant reduction of symptoms despite the persistence of some discharge from the external opening [9, 29]

A pooled rate of complications of 8% was reported for FiLaC in a systematic review and meta-analysis by Frountzas et al. [51]. The risk of continence disturbance is thought to be low following FiLaC. A high rate of 39% continence disturbance including both cases of mild incontinence for flatus as well as moderate incontinence for liquid stool after laser ablation of fistula tract was reported by Stijns et al. [31]. This extremely high rate of continence disturbance, even for conventional surgery, is contrary to almost all other series reported so far. For example, the systematic review on FiLaC for Crohn’s fistula by Cao et al. reported no single case of continence disturbance in all 50 patients who underwent surgery [47]. Similarly, De Bonnechose et al. [67] reported no single case of continence disturbance after FILaC in their study of 100 cases. An intriguing finding in their study was that no worsening of continence was seen in 15 patients with preoperative mild incontinence (failure to control flatus) [67].

R23:* Postoperative pain, risk of complications (especially continence disturbance), and healing rate are key outcome criteria following FiLaC.*

### Q24: Can FiLaC be repeated in case of failure?

Healing rates of 70% have been reported for Re-FiLaC [9, 63]. In a retrospective study from Germany, Wolicki et al. reported a healing rate of 78.3% (68 of 82 patients) after re-FiLaC [30]. In the systematic review by Elfeki et al., the cumulative healing rate after the second FiLaC was 69.7% [8]. The current literature, therefore, supports Re-FiLaC after an initial failure. According to expert opinion, the potential increase in healing rate, associated with the safe nature of the procedure, if correctly performed, represents a strong argument in favor of Re-FiLaC.

R24:* FiLaC can be repeated after an initial failed attempt.*

### Q25: What is the optimal follow-up after FiLaC?

So far, there is no recommended follow-up schedule after FiLaC. Therefore, an evidence-based follow-up recommendation cannot be made. Follow-up should be according to surgeon’s preference and should take local healthcare standards including availability of resources into consideration.

A follow-up schedule based on expert opinion may look like:

- Postop day 7: interview, inspection
- Postop weeks 2–6: interview, inspection, digital exam, anoscopy
- 3 months, 6 months, 12 months, 24 months, 36 months, 60 months

R25:* Follow-up could include interview, inspection, digital exam, and anoscopy at reasonable intervals.*

## Discussion

Striking a good balance between risk of complications and success rate in anorectal fistula may be a challenge. Tilting this balance toward acceptable success rates with a low risk of complication has been defined as an optimal outcome for patients undergoing fistula closure. This intricate balance becomes even more difficult to strike in cases with complex fistulae due to a high risk of sphincter injury. Amongst many outcome measures, fistula healing and continence represent two relevant outcome measures in a patient’s perspective. Lying open of simple fistulae almost always leads to healing without any relevant risk of continence disturbance. To reduce the risk of sphincter damage and hence risk of incontinence, sphincter-saving techniques have been employed in the management of complex fistulae. Following its first description in 2011 by Arne Wilhelm, the FiLaC procedure has been increasingly used and described as a sphincter-preserving technique for the closure of fistula in ano.

In the FiLaC procedure, laser energy is delivered by a radial probe to destroy and obliterate the fistula tract. This aspect of the procedure is probably the most consistent aspect of the FiLaC procedure so far. The current literature and clinical practice are characterized by huge heterogeneity in virtually all aspects of the procedure.

Success rates from as low as 20% up to as high as 80% have been reported for FiLaC [31, 68, 75]. One single aspect that is identical to most of the current publications on FiLaC is the retrospective study design. Differences in the current literature on FiLaC can be identified across almost every aspect of the procedure, beginning with patient selection, laser setting, and the execution of the procedure [38]. Therefore, the need for a standardized treatment protocol could not be overemphasized [11].

The RDG aimed to cover all relevant aspects of the FiLaC procedure. The RDG acknowledges the fact that some form of formal training is required before performing FiLaC. Thus, besides being trained in proctology, a workshop attendance, preferably with hands-on training, represents the minimum requirements prior to performing FiLaC. Moreover, the RDG strongly recommends some form of mentoring during the initial phase of the learning curve. Regarding the laser setting, the RDG reached very strong consensus on a radial emitting probe at 1470 nm wavelength and 12 W setting.

The preoperative aspects of these recommendations are basically in line with the current practice, especially with regard to the need for conditioning or cleaning the fistula tract by using a draining seton as well as preoperative imaging prior fistula closure [6, 76]. Lay-open of “simple” fistulae is generally recommended in the European and American guidelines owing to high healing rates with low risk of continence disturbance. While the RDG acknowledges the fact that fistulotomy has a higher healing rate in comparison to FiLaC, the minimally invasive and tissue-preserving nature of the FiLaC technique were seen as strong arguments in favor of this technique, in selected cases with simple fistulae (see Q8). Moreover, FiLaC can be repeated in case of failure, with an even higher success rate [8, 30, 51]. From a patient’s perspective, low postoperative pain, almost zero risk of incontinence, and reduced symptoms even in cases of failure argue in favor of FiLaC [9, 29].

Consensus was reached on all 25 points during the first voting round, with all but for two items (Q16 and Q17) scoring over 82% agreement. Combining FiLaC with an additional procedure was addressed in Q16. Considering FiLaC as a stand-alone procedure, adding a second technique to FiLaC automatically creates a confounder. This debate has been going on since the initial publication by Arne Wilhelm, who used an advancement flap to complement FiLaC [7]. In recent years, closing the internal opening with a simple stitch (Z-stitch or finger of eight stitch) has been shown to improve success rate. Similar to using a flap, it remains questionable which aspect of the surgery, FiLaC or closure of the internal opening, contributed to what degree of the success of the procedure. Despite these discussions, the current literature provides some guidance to this debate, so that closure of the internal opening with a simple stitch was recommended by the RDG. Question 17 looked at the shape and diameter of the internal opening as a possible determinant of success following FiLaC based on a single report that a large diameter of the internal opening may lead to low closure rate [67]. Discussion amongst experts in the RDG revealed relevant technical limitations in the cited work including the arbitrary nature of measuring the size of the internal opening (estimated), widening of the tract via curettage, and omission of closure.

## Limitations

First, the evidence level on which these recommendations are based is low to very low. The FiLaC literature, at least at this moment, consists largely of retrospective studies with well-known flaws. Second, there is a possibility that some relevant publications were missed by our search strategy. This is especially the case for studies published in foreign languages, since only studies published in English were included in this work. Third, even though the Delphi procedure was followed, the effect of “expert opinion” during discussion to reach consensus cannot be totally excluded.

Despite these limitations, we are confident that these recommendations would help standardize the FiLaC procedure, and at least to some extent increase the safety of the procedure. Moreover, we have identified and discussed crucial aspects of the procedure, identifying potential areas of future research, and at the same time provided a tool to enable comparison amongst different institutions and perhaps guide future research.

## Conclusion

The RDG offers a comprehensive suite of recommendations to enhance the safety and efficacy of standard FiLaC procedures. Out of 25 detailed recommendations, collectively addressing the full spectrum of FiLaC procedures—from laser settings and preoperative preparations to perioperative strategies and postoperative care. This coherent framework is anticipated not only to standardize but also to refine the FiLaC technique across the board, thereby elevating the management of fistula in ano. 

## Data Availability

No datasets were generated or analysed during the current study.
